# Characterization of Sub-Optical-Wavelength Structures through Optically Opaque Films Using Picosecond Ultrasonics

**DOI:** 10.1021/acs.nanolett.5c00800

**Published:** 2025-05-26

**Authors:** Maksym Illienko, Komal Chaudhary, Matthias C. Velsink, Stefan Witte

**Affiliations:** † 530573 Advanced Research Center for Nanolithography, Science Park 106, Amsterdam, 1098 XG, The Netherlands; ‡ Department of Imaging Physics, Faculty of Applied Sciences, 2860 Delft University of Technology, Lorentzweg 1, Delft, 2628 CK, The Netherlands

**Keywords:** ultrafast photoacoustics, subwavelength structures, acoustic diffraction, optical metrology, defect detection, picosecond ultrasonics

## Abstract

Periodic arrays of nanostructures form an important building block of modern integrated circuits and photonic devices. Functionality of such devices is often critically dependent on the detailed structure. Moreover, multistep lithographic processing requires accurate metrology tools to characterize device morphology noninvasively, often after the deposition of additional layers of material. Here we show that ultrafast picosecond ultrasonics enables the accurate characterization of periodic structures below optically opaque thin films. By optically generating and detecting ultrahigh-frequency ultrasound at the surface of the film, we quantitatively characterize the main features of subsurface gratings with line widths down to 100 nm. We find that the acoustic diffraction is sensitive to the shape of the grating lines on the scale of tens of nanometers.

## Graphical abstract

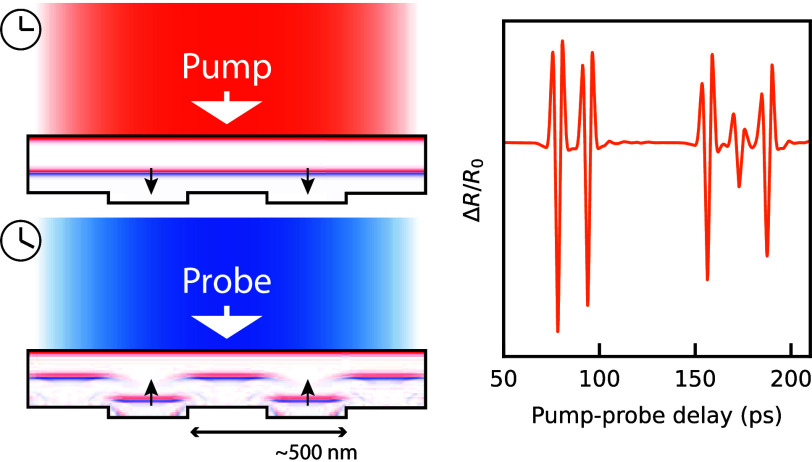

Lithographic fabrication of nanostructures is the core technology driving integrated circuit (IC) manufacturing. The miniaturization of electronics and the development of increasingly powerful computing systems are made possible by continuous improvements in IC technology. Modern IC designs feature complex 3D nanostructures, which require extremely precise lithography. Reliable manufacturing is therefore critically dependent on sub-nanometer-level positioning accuracy, as well as measurement methods to quantitatively characterize fabricated nanostructures.^1,2^ This field of semiconductor metrology involves characterization of the fabricated nanostructures in between lithography steps and on the completed devices but also covers methods that ensure accurate positioning of the silicon wafers before printing and position verification of the printed features relative to previously printed layers. Such methods are known as wafer alignment and overlay metrology and are typically performed on dedicated markers printed near the actual devices.^2^ These markers are typically periodic structures such as diffraction gratings, as they have a distinct and efficient optical response. In semiconductor metrology, optical methods are preferred because they are fast and noninvasive. However, as structures become much smaller than an optical wavelength and IC designs increasingly contain materials that are optically opaque, such optical methods are reaching their limits in terms of resolution and contrast. While alignment and overlay metrology can, in principle, be performed on larger structures that are optically detectable, their accuracy improves for smaller feature sizes. Importantly, as metrology markers are typically printed in the first lithography step, they may become covered by large amounts of material during the fabrication of complex multilayer devices such as 3D-NAND memory. To realize the ambitious scaling of IC device size and complexity pursued by the semiconductor industry, the development of new metrology concepts that are effective for optically opaque 3D nanostructures becomes crucial.^1^

In this work, we demonstrate the potential of ultrafast-laser-driven photoacoustics, also known as picosecond ultrasonics, for metrology on sub-optical-wavelength markers, through layers of optically opaque material. When a femtosecond laser pulse strikes a partially absorbing surface, the interaction with electrons and subsequent electron–phonon coupling results in the generation of coherent acoustic phonons with extremely high frequencies, approaching the THz range.^3–8^ These coherent acoustic phonons form a localized wavepacket that travels into the material at the speed of sound. Upon propagation, internal interfaces and structures give rise to reflection and diffraction.^6,9,10^ As such high-frequency wavepackets can contain wavelength components well below 100 nm, and efficient diffraction from structures in the 10–100 nm range can occur. When the diffracted wavepackets return to the surface, they can be characterized optically through their influence on the surface reflectivity, using a time-delayed probe laser pulse.^4,8,11^

Picosecond ultrasonics has been used to study thin-film layer properties,^10,12–15^ for the detection of buried structures,^16,17^ and as a contrast mechanism for microscopy.^18–21^ We now show that the technology can be extended to characterize far-sub-optical-wavelength structures by analyzing the temporal structure of the diffracted wavepackets that return to the surface.

We perform a series of experiments in which we generate and detect such acoustic pulses inside layers of zirconium with different periodic nanostructures patterned on the back surface. We detect the reflected and diffracted ultrasound wavepackets and retrieve the properties of the nanostructures from these signals. By comparing the experimental observations to simulation results, we can retrieve structural parameters and nanoscale shape variations of the markers.

The concept of picosecond ultrasonics on nanoscale buried periodic markers is shown in Figure 1. The sample is a uniform layer of opaque material with an etched grating on the back surface. The thickness of the layer and the pitch of the grating are on the order of several hundreds of nanometers. For clarity, we refer to the etched and unprocessed areas of the grating as lines and valleys, respectively. Pump and probe pulses from two separate but electronically synchronized lasers are focused into micron-sized spots onto the flat top surface of the sample (Figure 1a). The relative time delay between pump and probe pulses is scanned electronically, and probe reflectivity is detected with shot-noise-limited sensitivity through balanced detection and lock-in amplification.^22^ Details of the experimental setup are given in the Supporting Information (SI) and Figure S2.

**Figure 1:**
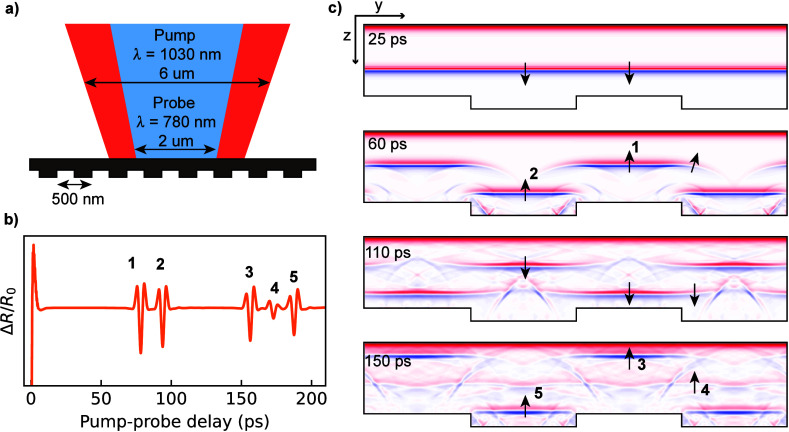
Concept of photoacoustics on nanoscale buried gratings. a) Cross-section sketch of the interaction with pump and probe beams. b) Simulated reflectivity change vs time. c) Four consecutive snapshots of the simulation of light-induced strain waves in the structure (an animation is available in the SI). The longitudinal strain component *ε*_*zz*_ is displayed, with red and blue colors indicating positive and negative strain, respectively. Black arrows indicate the direction of wave propagation. At 25 ps, a planar strain pulse is traveling toward the back surface of the sample. At 60 ps, two groups of strain pulses are seen returning to the surface, resulting from splitting of the initial planar pulse by the first reflection from grating lines and valleys. Numbered arrows correspond to the peaks in the reflectivity change. One can also see the lateral diffraction of the strain pulse, indicated by a tilted arrow. At 110 ps, the first group of pulses reaches the grating line again. Due to lateral broadening, part of the strain pulse couples into the valleys. In the same way, the second group of pulses partially reflects the lines. As a result, after the second reflection from the grating (150 ps), there is an additional strain pulse labeled by arrow 4. The constant strain at the top surface of the sample is caused by thermal expansion of the sample.

Measuring probe reflectivity within a certain pump–probe time-delay range gives a specific reflectivity change pattern determined by the buried grating (Figure 1b). Two-dimensional finite-difference time-domain (FDTD) simulations of the generated and propagating strain profiles^23^ are shown in Figure 1c for different times after excitation by the pump pulse. In the chosen geometry, where the optical spots are significantly larger than the grating period and the probe spot is smaller than the pump (see Figure 1a for typical numbers), we can consider homogeneous acoustic excitation and detection across the probe spot. Such a geometry allows simulation of a single grating period with homogeneous pump excitation and periodic lateral boundary conditions. The numerical simulation approach is explained in detail in the SI section Theoretical model.

As illustrated in Figure 1c, the pump pulse initially induces a planar longitudinal strain pulse that propagates toward the buried grating. Upon reflection from the grating, two time-separated groups of strain pulses are formed by the reflection from the lines and valleys (marked by 1 and 2). In addition, diffraction from the line edges leads to curved wavefronts around those edges. Reflection from the top surface leads to a second roundtrip of the strain pulses, which is largely a copy of the first roundtrip, with one important difference: the diffracted part of the acoustic strain wave from the line now couples into the space, and vice versa. The expected normalized reflectivity change *ΔR*/*R*_0_ at the surface is plotted in Figure 1b. Five distinct peaks can be identified, which correspond to the different strain wave components from lines and valleys returning to the surface, as given in Figure 1c. Whereas peaks 1,3 and 2,5 correspond to the first and second roundtrips on lines and valleys, peak 4 results from the line edge diffraction described above. This ‘diffraction echo’ is specifically sensitive to the shape of the grating lines, as will be shown below.

The time dependence of *ΔR*/*R*_0_ can thus be used to retrieve the structural properties. As the roundtrip time delay encodes layer thickness^10,12,14^ and local thickness variation,^18^ the time delay between peaks 1 and 2 encodes grating line height. If the wavelength of the strain wave is much shorter than the grating line width, the ratio of signals 1 and 2 can directly be interpreted as the duty cycle of the grating, i.e., the relative width of lines and valleys. However, as the finite acoustic wavelength leads to diffraction, this also affects the reflected wave from the lines and valleys in a different way. As can already be seen at *t* = 60 ps in Figure 1c, edge diffraction in the valley leads to part of the strain pulse being strongly diffracted, which results in a reduction of the signal returning from the valley relative to the line, such that this second peak will be weaker than the first for a grating with 50% duty cycle. Through this mechanism, the detected signal is sensitive to the pitch of the grating. In addition, the grating pitch determines the relative amount of coupling of strain waves between lines and spaces. This effect makes the amplitude of the diffraction peak dependent on the grating pitch.

Given this sensitivity to grating parameters, a logical question is whether quantitative retrieval of those parameters from the measured time-dependent *ΔR*/*R*_0_ signals is possible. A complicating factor for such quantitative retrieval is that in addition to the grating parameters the resulting *ΔR*/*R*_0_ signal is strongly influenced by the elastic, optical, and thermodynamic properties of the material. These properties determine the process of strain pulse generation and propagation, as well as the sample’s optical response to a given strain distribution near the top surface. However, if these material properties are known, numerical simulations of the photoacoustic interactions^23,24^ can quantitatively predict the time-dependence of *ΔR*/*R*_0_ in the presence of a grating structure. Such models can then be used to solve the inverse problem of retrieving the structural properties of the buried features. The accuracy of such a procedure naturally depends on the completeness of the model and the quality of the data but especially on the sensitivity to the nanoscale structural properties. To address these aspects, we performed systematic experiments and simulations of a range of different nanostructures.

For the experiments, a series of 10 × 10 μm gratings with various pitches and duty cycles were milled in the 400 nm thick zirconium freestanding membrane with the use of a gallium focused ion beam (FEI Helios NanoLab 600). A typical photoacoustic response is shown in Figure 2a. For reference, a measurement at an unstructured part of the Zr membrane is shown as the blue trace, showing two clear echo signals that correspond to consecutive roundtrips of the acoustic wavepacket. The orange trace is the result of a measurement at the location of a grating with 600 nm pitch and 50% duty cycle. The measurement clearly shows the five expected peaks, as discussed above. A scanning electron microscope (SEM) image of the grating, recorded from the patterned side, is shown in Figure 2b.

**Figure 2:**
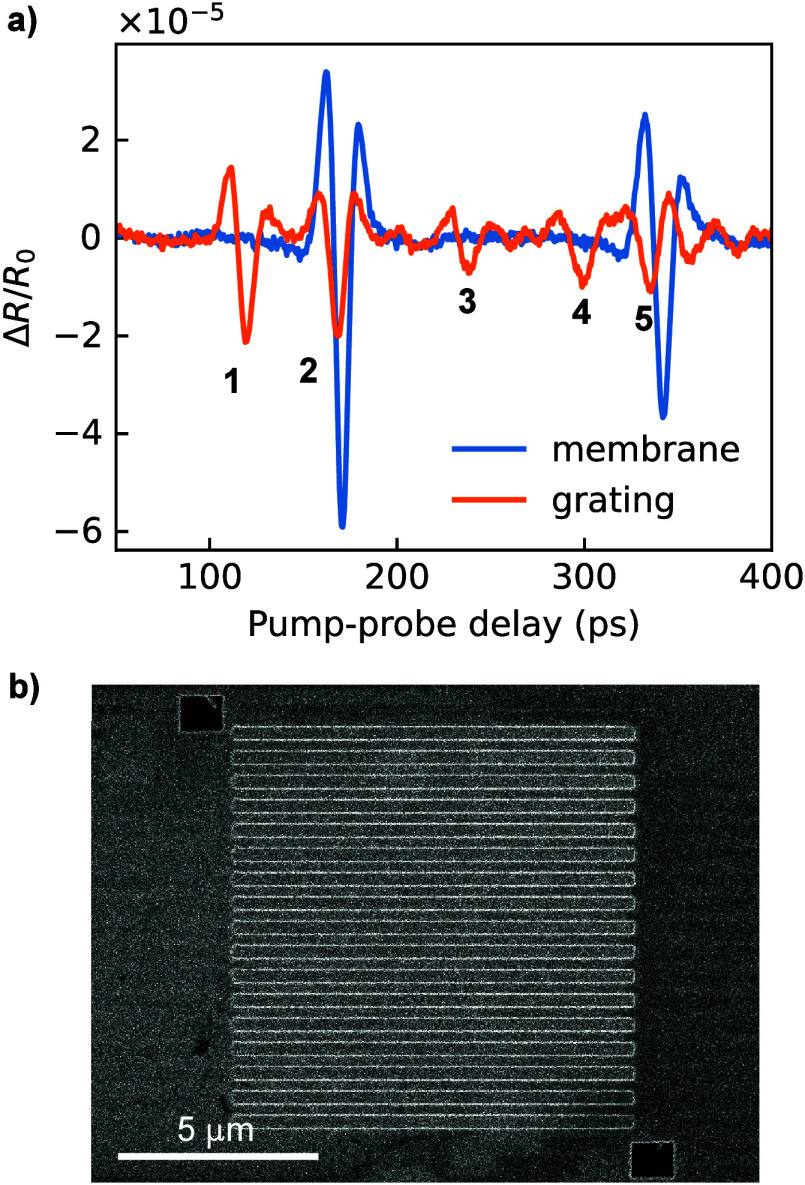
a) Experimentally observed time-dependent reflectivity variation for a flat 400 nm thick freestanding membrane of Zr and the film with imprinted 600 nm pitch and 50% duty cycle grating. Initially, measurements contain a significant thermal background that is caused by nanosecond-scale temperature dynamics in the sample. This thermal background is removed by 15 GHz high-pass filtering (for more details see the SI section on the fitting procedure). Five peaks are visible in the signal from the grating, which correspond to the expectation from the simulations. b) SEM image of a 600 nm pitch grating milled into a Zr film.

To retrieve the required photoacoustic properties of zirconium, we first fit the measurements taken on the unprocessed membrane to our numerical model (see the SI section on the fitting procedure and Figure S3). The retrieved material parameters are then used to simulate the expected measurement response in the presence of grating structures. In Figure 3 we show measured and simulated reflectivity curves for 600 and 400 nm pitch gratings, both with a 50% duty cycle. The simulations show a clear sensitivity to the structural properties, and the best match between simulation and experiment is found for grating line widths of 264 and 164 nm, respectively. A further analysis of the sensitivity of these experiments to pitch and duty cycle is given in SI Figure S4. The observed correspondence between simulations and measurements shows the ability to quantitatively determine the structural properties of such buried nanostructures using picosecond ultrasonics.

**Figure 3:**
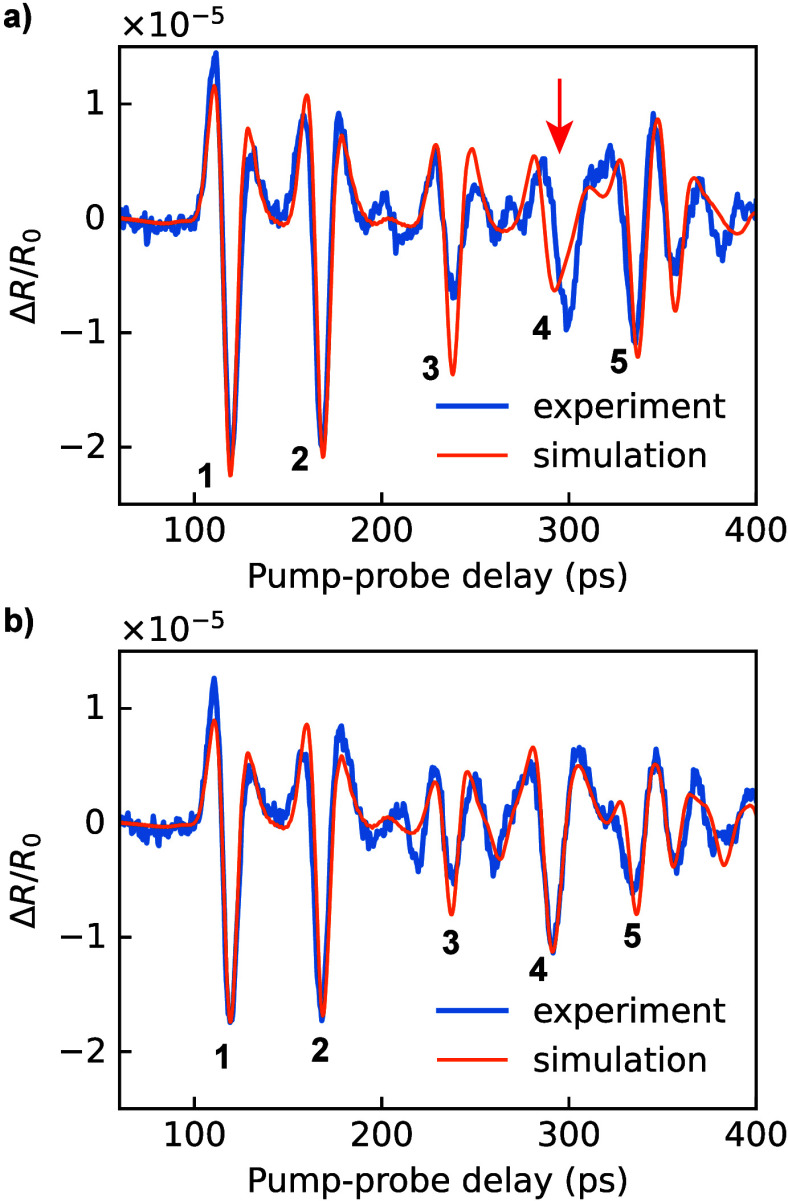
Experimental results and simulations for 50% duty cycle gratings with a) 600 and b) 400 nm pitch. The red arrow highlights the diffraction peak, of which the timing is influenced by the grating line shape (see text for details).

However, in the results of Figure 3 some discrepancy between theory and measurements remains. In particular, the timing of the diffraction echo (peak 4) does not match the expectation for the 600 nm pitch grating, and simulations show that this timing is not sensitive to the grating pitch and line width (see SI Figure S4). As this particular peak originates from acoustic diffraction at the line edges, we therefore hypothesize that the diffraction echo shape and timing are sensitive to the exact shape of the buried features. While the simulations shown in Figure 3 assumed grating lines with a rectangular profile, the finite spot of the focused ion beam and redeposition effects during the milling process typically result in a nonrectangular profile of the fabricated patterns.

To investigate this possibility, we measured the *ΔR*/*R*_0_ response of multiple gratings with varying parameters and performed simulations that include varying line shapes, which we compared to SEM cross sections of the gratings. We fabricated a set of 600 nm pitch gratings with nominal line widths of 100, 200, and 300 nm, as well as 400 and 800 nm pitch gratings with a 50% duty cycle. All gratings were fabricated on the same freestanding Zr membrane. The resulting signals are shown in Figure 4a, together with the fit results. As a reference for the line shape model, side-view images of the fabricated structures were recorded by milling a rectangular aperture through the membrane near the side of each structure and taking an SEM image at an oblique angle (Figure 4b). The oblique SEM viewing angle did not allow a quantitative comparison, as the exact viewing angle could not be calibrated, and the resolution was limited in this configuration. Nevertheless, these images clearly show more rounded line shapes, with significant variations for different line widths, and consistent sidewall angles.

**Figure 4:**
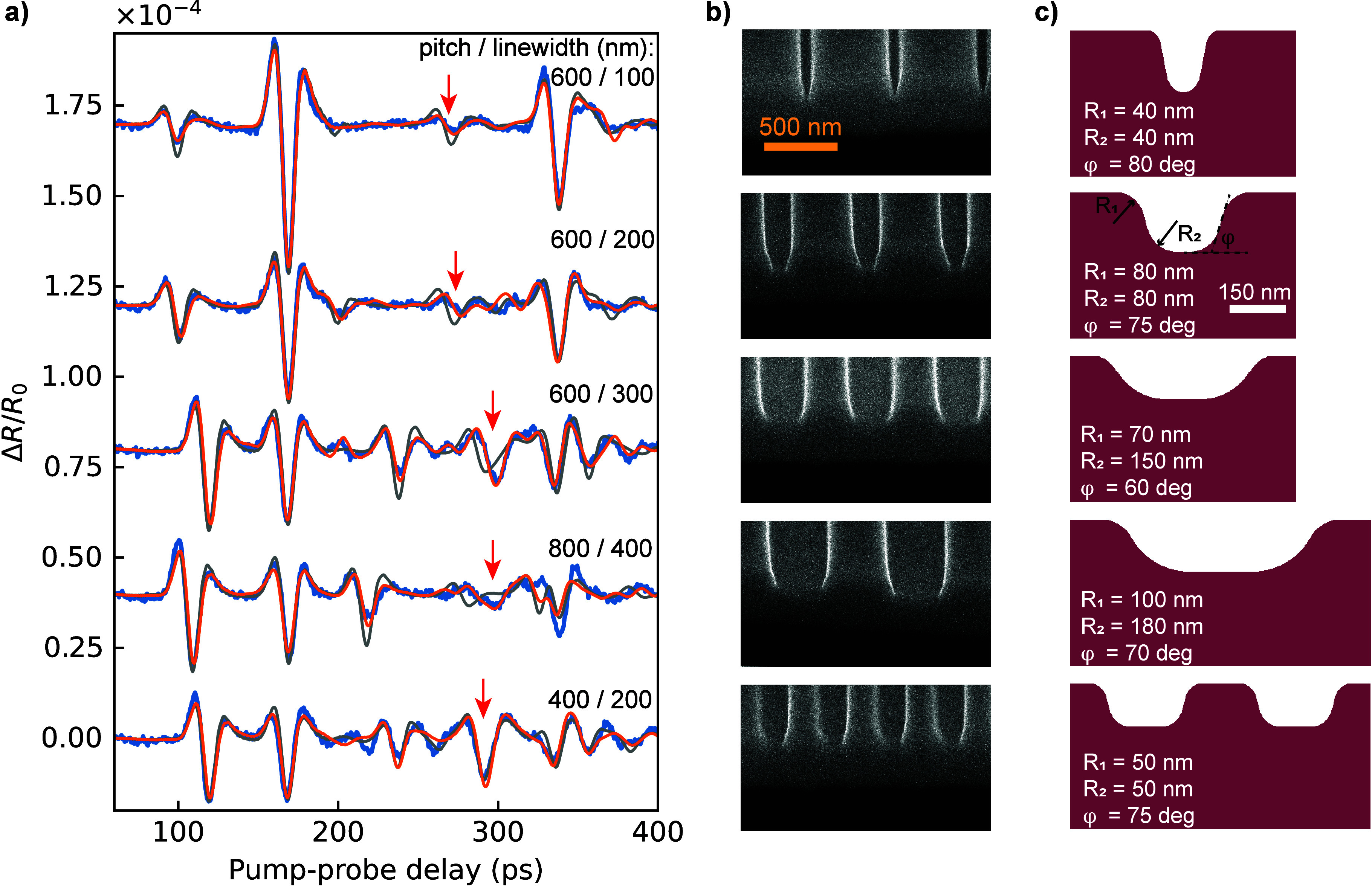
Sensitivity to nanoscale shape variations. a) *ΔR*/*R*_0_ traces for various values of pitch and duty cycle. Blue curves represent experimental measurements, gray, best-matching simulations with rectangular line shapes, and orange, simulations with rounded line shapes. Orange curves show reasonably better agreement with the experimental measurements, especially around the diffraction peak (depicted by red arrows). b) SEM images of gratings cross sections taken at an oblique angle. Significant deviations from the rectangular shape of the grating lines are observed. c) Rounded profiles of grating lines used to simulate orange traces in a).

To assess the sensitivity of the photoacoustic response to such shapes, we expand the simulations to model the grating sides with a smoother line shape consistent with the shapes seen in the SEM images (see the SI section on sensitivity to grating pitch, duty cycle, and line shape and Figures S4–S6 for more details). This line shape is an inverted isosceles trapezoid with rounded corners which is parametrized by base angle and radii of top and bottom curvatures. Figure 4c shows the optimized shape for each grating, while the corresponding simulated *ΔR*/*R*_0_ signals for all of these structures are overlaid with the measured data in Figure 4a. For comparison, the gray traces correspond to the best-matching simulations when assuming rectangular line shapes. For all signals, the improvement resulting from the optimized line shapes is clear, especially around the diffraction echo, which is indicated by the red arrow in each trace. A general observation is that the simulated signals using the optimized line shape accurately predict the timing of the diffraction echo. Thus, the measured time dependence of *ΔR*/*R*_0_ indeed contains sufficient information to determine both the grating parameters and the line shapes. Overall, very good agreement is obtained between the simulation and experiment. This agreement persists even for the narrowest lines used in these experiments with a width of only 100 nm. Using a parametrization of the nanostructures, such as the rounded trapezoid-type line shapes discussed above, enables a fitting procedure to determine the line shape that best matches the experimental data (see SI Figure S6). We find that other parametrizations can be used in a similar way, such as a sigmoidal line shape (see Figure S7).

From the analysis of the present experiments and a comparison with simulations for a range of different grating parameters (see Figures S4–S6), we conclude that we can retrieve pitch, line width, and line shape features with a sensitivity in the range of tens of nanometers. It is worth noting that this has been achieved using infrared light with a wavelength of 780 nm, which exceeded the pitch of the detected gratings, and optical spot diameters of 2–4 μm. The sensitivity to nanoscale structural features results from the extremely short acoustic wavelength propagation in the material. In the case of Zr, the central wavelength of the optically generated acoustic wavepacket was around 150 nm. The frequency content of the thermoelastically generated phonon wavepacket is mainly determined by the optical absorption depth and electron transport properties of the material. In many materials, particularly optically opaque ones, such high-frequency phonon wavepackets can then be generated with appropriate pump beam properties. Therefore, picosecond ultrasonics has significant potential as a metrology tool for optically opaque nanostructures.

In the present work, the measured *ΔR*/*R*_0_ signals were averaged over the spatial scale of the probe spot, which, in principle, limits the reconstruction ability to either periodic or isolated nanostructures. However, picosecond ultrasonics can be expected to be sensitive to defects in a perfect periodic structure as well, as such defects affect the acoustic diffraction as well. To explore this possibility, we performed simulations in which we introduce defects into a periodic pattern. Three different types of defects are investigated, as shown in Figure 5a, where a single grating line is missing completely, 50% narrower, or 50% shallower than intended. In all cases, we assume an FWHM probe spot diameter of 2 μm. Figures 5b–d show the resulting reflectivity signals compared to the defect-free case. Each of these defects leads to a distinct difference in signal, with a magnitude that can readily be detected with the measurement sensitivity of our system. Importantly, the defect-induced signal shape cannot be mimicked by a change in the various fit parameters. Examples of such parameter adjustments are shown in Figures 5e,f, where a fit was attempted to a defect-free grating with an adjusted duty cycle to correct for the defect-induced signal shape change. While such a parameter adjustment can correct for the height of some of the echoes (in this case, the first two), the fit quality is significantly reduced. We therefore conclude that picosecond ultrasonics can be used to identify the presence of defects in nanoscale structures, as well. A smaller probe spot would sample a reduced number of grating lines, which would further increase the sensitivity to nanoscale defects and isolated structures. Using more advanced parametrization, it should be feasible to characterize the shape of the defects. By developing more advanced measurement concepts based on, for example, detailed sampling of the acoustic diffraction as a function of spatial position, we expect that the approach extends to more complex 3D nanostructures as well.

**Figure 5:**
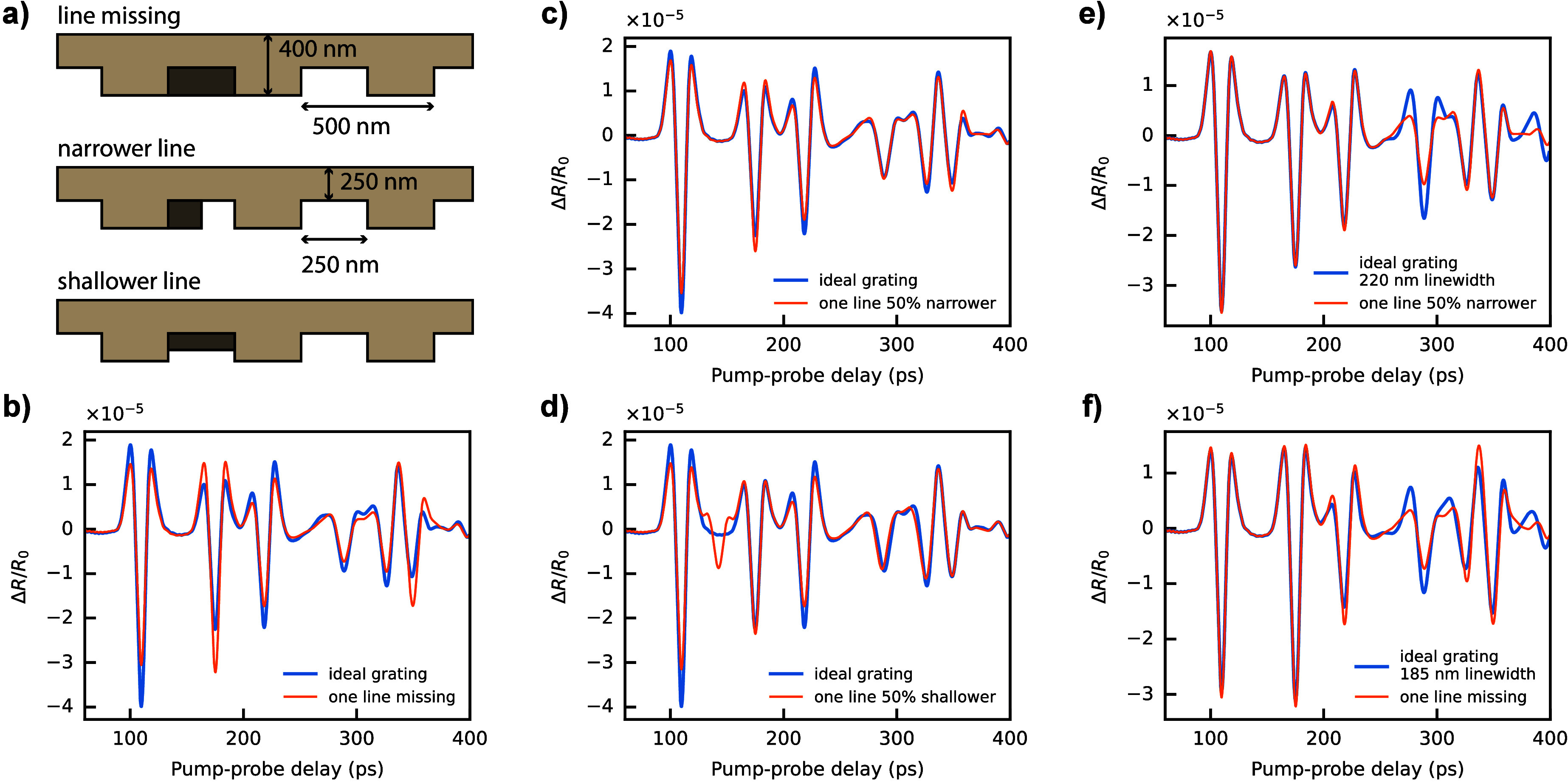
Sensitivity to grating defects. a) Schematics of the simulated gratings with defects. b–d) Comparison of reflectivity curves obtained from the ideal grating and the grating containing a defect. e, f) Comparison of signals from gratings with defects and ideal gratings with adjusted line width.

## Supplementary Material





## Notes

Video animation of light-induced strain wave propagation in the patterned membrane (MP4)
Background information on the simulations and underlying theory, the procedure for fitting to experimental data, and an analysis of the signal sensitivity to the main grating parameters (PDF)
