# Supplementary material for: Characterization of Sub-Optical-Wavelength Structures through Optically Opaque Films Using Picosecond Ultrasonics
Source: Nano Lett. 2025 May 26;25(22):8909–14. doi: 10.1021/acs.nanolett.5c00800 (PMC12142679; doi:10.1021/acs.nanolett.5c00800)
Supplement: Supplementary file 2 [file nl5c00800_si_002.pdf]

Supporting Information for

Characterization of sub-optical-wavelength structures through optically opaque films using picosecond ultrasonics

Maksym Illienko[1], Komal Chaudhary[1], Matthias C. Velsink[1], and Stefan Witte*[1,2]

[1]*Advanced Research Center for Nanolithography, Science Park 106, Amsterdam, 1098 XG, The Netherlands*
[2]*Department of Imaging Physics, Faculty of Applied Sciences, Delft University of Technology, Lorentzweg 1, Delft, 2628 CK, The Netherlands*

Theoretical model

In the current work, photoacoustic experiments were conducted on a patterned zirconium freestanding membrane. The process of acoustics generation in metals can be sufficiently described by the thermoelastic effect [1, 2] with a combination of the Two Temperature Model (TTM) [3,4]. Upon absorption, the picosecond pump pulse transfers its energy into the electron subsystem that undergoes a rapid temperature increase while the lattice subsystem remains at environmental temperature. Then, electrons thermalize with lattice on the picosecond time scale via the electron-phonon coupling. To describe this process, we use hyperbolic TTM [5]:

$$
\begin{aligned}
C_e(T_e)\frac{\partial T_e}{\partial t} + \nabla \cdot \mathbf{Q}_e &= -G(T_e - T_l) + S(x, y, z, t);\\
\tau_e \frac{\partial \mathbf{Q}_e}{\partial t} + \mathbf{Q}_e &= -k_e(T_e, T_l)\nabla T_e;\\
C_l \frac{\partial T_l}{\partial t} &= G(T_e - T_l).
\end{aligned}
\tag{1}
$$

Where T_e and T_l are the electron and lattice temperature respectively, $\mathbf{Q}_e$ is the vector of electron heat flux, C_e and C_l are the electron and lattice heat capacity, k_e is the electron thermal conductivity, τ_e is the electron relaxation time, G is the electron-phonon coupling constant, and S is the source defined by pump light absorbed power density. We consider temperature dependences of $C_e = \gamma T_e$ and $k_e = k_{eq} T_e / T_l$ [5–7]. The electron relaxation time is theoreticaly calculated as $\tau_e = 3k_e / v_F^2 C_e$ [6], where v_F is the Fermi velocity. The temperature dependence of τ_e is neglected. The absorbed power density is calculated with the transfer matrix method [8] assuming the Gaussian temporal profile of the pump pulse.

Change of lattice temperature ΔT_l gives rise to thermal stress which in the case of isotropic medium is a diagonal tensor:

$$\sigma_{ij}^{th} = -3K\alpha\delta_{ij}\Delta T, \tag{2}$$

where K is the bulk modulus, α is the thermal expansion coefficient, δ_{ij} is the Kronecker delta tensor. Thermal stress induces elastic waves which we describe by employing the linear elasticity theory. It is governed by the

*smwitte@tudelft.nl

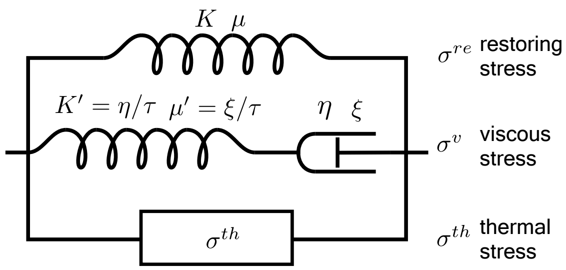

Figure S1: Schematics of the standard linear solid model. Total stress consits of restoring σ^{re}, viscous σ^{v}, and thermal σ^{th} components.

elastodynamics equation [9]:

$$\rho \frac{\partial v_i}{\partial t} = \frac{\partial \sigma_{ik}}{\partial x_k}, \tag{3}$$

and by the Hooke's law (isotropic medium) [9]:

$$\sigma_{ij} = K\varepsilon_{ll}\delta_{ij} + 2\mu \left(\varepsilon_{ij} - \frac{1}{3}\varepsilon_{ll}\delta_{ij} \right). \tag{4}$$

Where v_i is the velocity vector, σ_{ik} is the stress tensor, ρ is the mass density, ε_{ij} is the strain tensor, K and μ are bulk and shear moduli respectively. Einstein summation is implied in Eq. 3 and 4. To include damping of elastic waves we use standard linear solid model shown in Fig. S1. In this model springs and dashpots represent elastic and viscous properties of medium respectively. The total stress consists of restoring stress σ^{re} caused by body deformation, viscous stress σ^{v} that emulates friction forces, and thermal stress σ^{th}. Stress in each spring in Fig. S1 is determined by Eq. 4 while stress in the dashpot is [9]

$$\sigma_{ij} = \eta\dot{\varepsilon}_{ll}\delta_{ij} + 2\xi \left(\dot{\varepsilon}_{ij} - \frac{1}{3}\dot{\varepsilon}_{ll}\delta_{ij} \right), \tag{5}$$

where η and ξ are viscous coefficients. Dot above the strain tensor depicts time derivative. From the Fig. S1 one can see that viscous stress response is noninstantaneous to the change of strain but characterized by the relaxation time τ. This leads to the relaxation damping behavior observed in picosecond photoacoustics experiments [10–13]. For a given geometry of a sample, we can consider a two-dimensional problem with y and z axes oriented along the grating vector and acoustics propagation respectively. Thus, ε_{yy}, ε_{zz}, and ε_{yz} are the only non-zero components of the strain tensor. In this case, Eqs. 2-5 applied to the model shown in Fig. S1 form the following system:

$$\begin{aligned}
&\sigma^{re}_{yy} = M\varepsilon_{yy} + \lambda\varepsilon_{zz}; && \sigma^{v}_{yy} + \tau\dot{\sigma}^{v}_{yy} = M_v\dot{\varepsilon}_{yy} + \lambda_v\dot{\varepsilon}_{zz};\\
&\sigma^{re}_{zz} = \lambda\varepsilon_{yy} + M\varepsilon_{zz}; && \sigma^{v}_{zz} + \tau\dot{\sigma}^{v}_{zz} = \lambda_v\dot{\varepsilon}_{yy} + M_v\dot{\varepsilon}_{zz};\\
&\sigma^{re}_{yz} = 2\mu\varepsilon_{yz}; && \sigma^{v}_{yz} + \tau\dot{\sigma}^{v}_{yz} = 2\mu_v\dot{\varepsilon}_{yz};\\
&\sigma^{th}_{yy} = \sigma^{th}_{zz} = -3K\alpha\Delta T_l; &&
\end{aligned}$$

$$\frac{\partial \varepsilon_{yy}}{\partial t} = \frac{\partial v_y}{\partial y}; \qquad \frac{\partial \varepsilon_{zz}}{\partial t} = \frac{\partial v_z}{\partial z}; \qquad \frac{\partial \varepsilon_{yz}}{\partial t} = \frac{1}{2}\left(\frac{\partial v_y}{\partial z} + \frac{\partial v_z}{\partial y} \right); \tag{6}$$

$$\begin{aligned}
\rho\frac{\partial v_y}{\partial t} &= \frac{\partial \sigma^{re}_{yy}}{\partial y} + \frac{\partial \sigma^{v}_{yy}}{\partial y} + \frac{\partial \sigma^{th}_{yy}}{\partial y} + \frac{\partial \sigma^{re}_{yz}}{\partial z} + \frac{\partial \sigma^{v}_{yz}}{\partial z};\\
\rho\frac{\partial v_z}{\partial t} &= \frac{\partial \sigma^{re}_{zz}}{\partial z} + \frac{\partial \sigma^{v}_{zz}}{\partial z} + \frac{\partial \sigma^{th}_{zz}}{\partial z} + \frac{\partial \sigma^{re}_{yz}}{\partial y} + \frac{\partial \sigma^{v}_{yz}}{\partial y}.
\end{aligned}$$

Where

$$M = K + \frac{4}{3}\mu; \qquad M_v = \eta + \frac{4}{3}\xi;$$
$$\lambda = K - \frac{2}{3}\mu; \qquad \lambda_v = \eta - \frac{2}{3}\xi; \tag{7}$$
$$\mu_v = \xi.$$

Equations 1 and 6 are solved numerically with proper boundary conditions. Since both pump and probe focus spots fit a significant amount of grating lines, we limit the simulation domain to a single grating period with periodic lateral boundary conditions for thermodynamic and elastic equations. The source S is assumed to be spatially homogeneous. For the front and back surfaces of the sample, we apply thermal-isolating and stress-free boundary conditions.

To calculate a reflectivity of the sample one needs to consider the perturbation of the dielectric permittivity caused by strain waves through the photoelastic effect. For isotropic medium and given geometry, the tensor of dielectric permittivity change is

$$\Delta\epsilon = -\epsilon^2 \begin{pmatrix} P_{12}(\varepsilon_{yy} + \varepsilon_{zz}) & 0 & 0 \\ 0 & P_{11}\varepsilon_{yy} + P_{12}\varepsilon_{zz} & P_{44}\varepsilon_{yz} \\ 0 & P_{44}\varepsilon_{yz} & P_{12}\varepsilon_{yy} + P_{11}\varepsilon_{zz} \end{pmatrix}, \tag{8}$$

where ϵ is the unperturbed dielectric permittivity, P_{11}, P_{12} and $P_{44} = (P_{11} - P_{12})/2$ are the photoelastic coefficients. One can see that under the strain wave medium becomes anisotropic. Furthermore, the orientation of anisotropy axes depends on the shear strain component ε_{yz}. An additional complication comes from spatial inhomogeneity of strain on the scale below the wavelength of the probe light. In general, one needs to solve Maxwell's equations to obtain the reflected field of the probe pulse. However, since the perturbation of dielectric permittivity is usually below $0.01\,\%$ and light propagation into the sample is limited by a couple of tens of nanometers, we approximate the reflected probe intensity by solving a one-dimensional problem at each y point with subsequent incoherent integration of reflected field over simulation domain. The solution of a one-dimensional problem is obtained with the transfer matrix method [8] which takes into account longitudinal inhomogeneities of the medium. From Eq. 8, one can conclude that the two eigenpolarizations are along the $x-$ and $y-$directions. Theoretical analysis shows that for both polarizations, in a first-order approximation the refractive index change is proportional to the ε_{yy} and ε_{zz} strain components. The shear component ε_{yz} only appears as the second-order term in the expression for the y polarization and thus can be neglected. With this approximation, calculations of probe reflectivity can be simplified further. The lateral periodic boundary conditions imply that there is a symmetry of the strain profile along the y axis. In particular, the integral of both components ε_{yy} and ε_{yz} within a period of the grating along y axis is zero, meaning that the ε_{yy} strain component does not contribute to a reflectivity change. For normal probe incidence, the change in dielectric permittivity can then be considered isotropic:

$$\Delta\epsilon = -\epsilon^2 P_{12}\varepsilon_{zz}. \tag{9}$$

Experimental setup

Experiments were performed with the use of the pump-probe technique via modulated asynchronous optical sampling [14]. The schematic of the setup is shown in Fig. S2. The pump (Menlo System Orange) and probe (Menlo System C-Fiber 780) are separated laser sources running at $50\,\mathrm{MHz}$ and $100\,\mathrm{MHz}$ repetition rate respectively. Repetition rates are electronically synchronized with a controlled offset allowing for the pump-probe delay tuning [14,15]. The pump pulses have central wavelength of $1030\,\mathrm{nm}$ and a pulse duration $180\,\mathrm{fs}$, while corresponding values for probe pulses are $780\,\mathrm{nm}$ and $80\,\mathrm{fs}$. Pump and probe beams are collinearly focused onto sample by a microscope

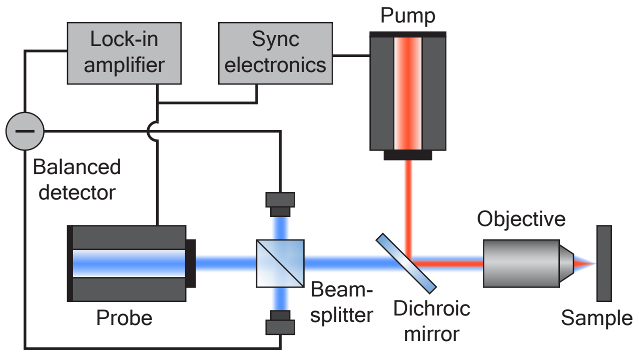

Figure S2: Schematics of the experimental setup.

objective (Olympus LUCPLFLN20X, x20, N.A. 0.45). The $1/e^2$ spot radii of the beams on the sample are $2.9\,\mu m$ and $1.0\,\mu m$ for pump and probe respectively. The reflected probe light is detected by a multimode fiber-coupled balanced detector ($500\,MHz$, Femto HBPR). The detector signal is then amplified by a lock-in amplifier (Zurich Instruments UHFLI) at $50\,MHz$ demodulation frequency.

The fitting procedure

Figure S3a shows the raw experimental data for a flat Zr membrane with a thickness of $400\,nm$. Significant reflectivity drop at time delay $t = 0$ is caused by the high temperature of the electron subsystem which undergoes instant heating upon pump pulse absorption. Within several picoseconds, the electron subsystem thermalizes with the lattice leading to strain waves generation and thermally induced reflectivity change. The thermal effect is observed as a slowly decaying background in Fig. S3a. Since only signals caused by strain waves are relevant, we remove the thermal background by applying a zero-phase high pass filtering with $15\,GHz$ cutoff frequency. The filtered signals are shown in Fig. S3b.

In order to perform simulations one needs to know the material parameters that are used Eqs. 1, 6, and 9. Some

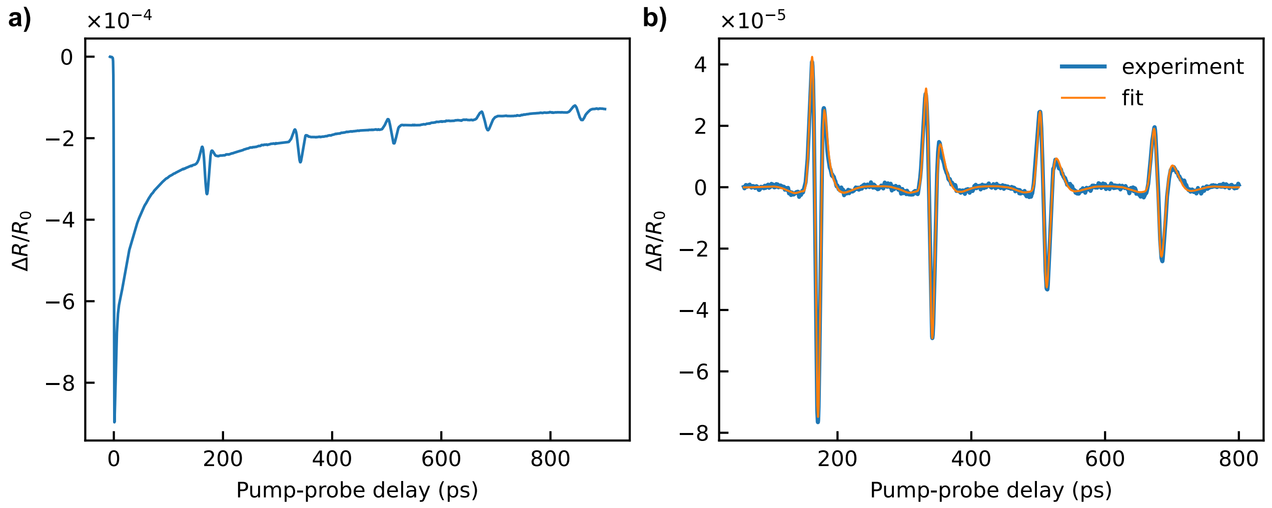

Figure S3: a) Experimental measurement performed on $400\,nm$ thick flat Zr membrane. b) Experimental curve after applying $15\,GHz$ high-pass filtering and best-fit simulation curve.

Table S1: Zirconium parameter values used in simulations

Parameter	Explanation	Value	Units	Source
γ	Electron volumetric heat capacity constant	89.5	$\mathrm{J/m^3K^2}$	$\gamma = \pi^2 n_e k_b^2 / 2E_F$ [6]
G	Electron-phonon coupling coefficient	7.74×10^{18}	$\mathrm{W/m^3K}$	$G = \pi^4 (n_e c_s k_b)^2 / 18 k_{eq}$ [5]
τ_e	Electron relaxation time	0.65	fs	$\tau_e = 3k_{eq}/C_e v_F^2$ [6]
k_{eq}	Electron heat conductivity	22.6	$\mathrm{W/mK}$	[16]
C_l	Lattice volumetric heat capacity	1.8×10^6	$\mathrm{J/m^3K}$	[17]
ρ	Mass density	6505	$\mathrm{kg/m^3}$	[17]
α	Thermal expansion coefficient	5.69×10^{-6}	$1/\mathrm{K}$	[17]
K	Bulk modulus	91.7	GPa	[17]
μ	Shear modulus	32.8	GPa	[17]
n_{pump}	Complex refractive index at 1030 nm	$4.16 + i3.84$	–	[18]

n_e – concentration of free electrons.
k_b – Boltzmann constant.
c_s – effective speed of sound obtained from Debye temperature.
E_F, v_F – Fermi energy and velocity respectively.

Table S2: Best-fit parameter values on 400 nm thick flat Zr membrane

Parameter	Explanation	Value	Units
n_{probe}	Complex refractive index at 780 nm	$4.47 + i3.26$	–
P_{12}	Complex photoelastic constant	$-0.021 + i0.128$	–
M_v	Viscous parameter	1.14×10^{-2}	Pa s
τ	Elastic relaxation time	2.46	ps

of these parameters are found in literature or theoretically estimated (Table S1). Others are fitted with the use of a non-linear least squares algorithm. Fitted parameters are the refractive index at probe wavelength, photoelastic constant, viscous coefficients, and elastic relaxation time. The fitting is performed on the measurements on a flat Zr membrane. Since the focus spots of the pump and probe are much bigger than the thickness of the membrane, we consider a one-dimensional problem for the fitting procedure. In this case, we can fit only one (M_v) out of two viscous parameters. For further simulations of the patterned membrane, we assume $M_v = \lambda_v$ i.e. $\mu_v = 0$. Values for fitted parameters are shown in Table S2.

Sensitivity to grating pitch, duty cycle, and grating lineshape

Figure S4 shows measured signals from a grating with a nominal pitch of 600 nm and 50 % duty cycle, together with simulation results for different pitches and duty cycles, assuming a rectangular profile of grating lines. In Fig. S4a, simulations with varying pitch are compared to the data, and it can be concluded that the sensitivity to pitch is limited, although a variation of 60 nm leads to a detectable difference in signal. Pitch mainly affects the amplitude of the diffraction peak. The sensitivity to duty cycle is much higher, having a strong effect on the relative height of peaks 1 and 2. Comparing simulation results to the experimental data, the 30 nm variations shown in Fig. S4b are

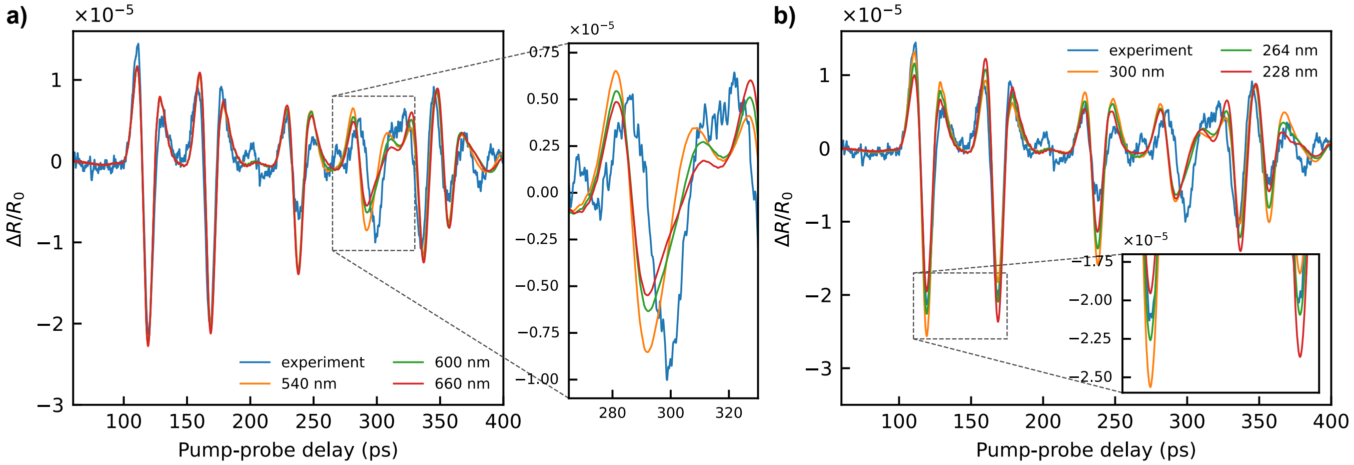

Figure S4: Dependence of the time-dependent reflectivity on pitch and linewidth of the grating. a) Simulated reflectivity change curves for different pitch (values indicated in the legend). The linewidth is fixed at $264\,\mathrm{nm}$. b) Simulated reflectivity change curves for different linewidths (values indicated in the legend). The pitch is fixed at $600\,\mathrm{nm}$.

readily discernible.

However, one can see from Fig. S4 that the discrepancy between theory and measurements can not be fully eliminated by adjusting only the pitch and linewidth of the grating. The disagreement is the strongest for the longer delay when the acoustics diffraction plays a significant role. A particular mismatch is the wrong timing of the diffraction peak highlighted in Fig. S4a. We hypothesize that the shape of the grating lines affects the shape of later echoes. The simplest modification of the grating lineshape is to introduce the inclination of the side walls of

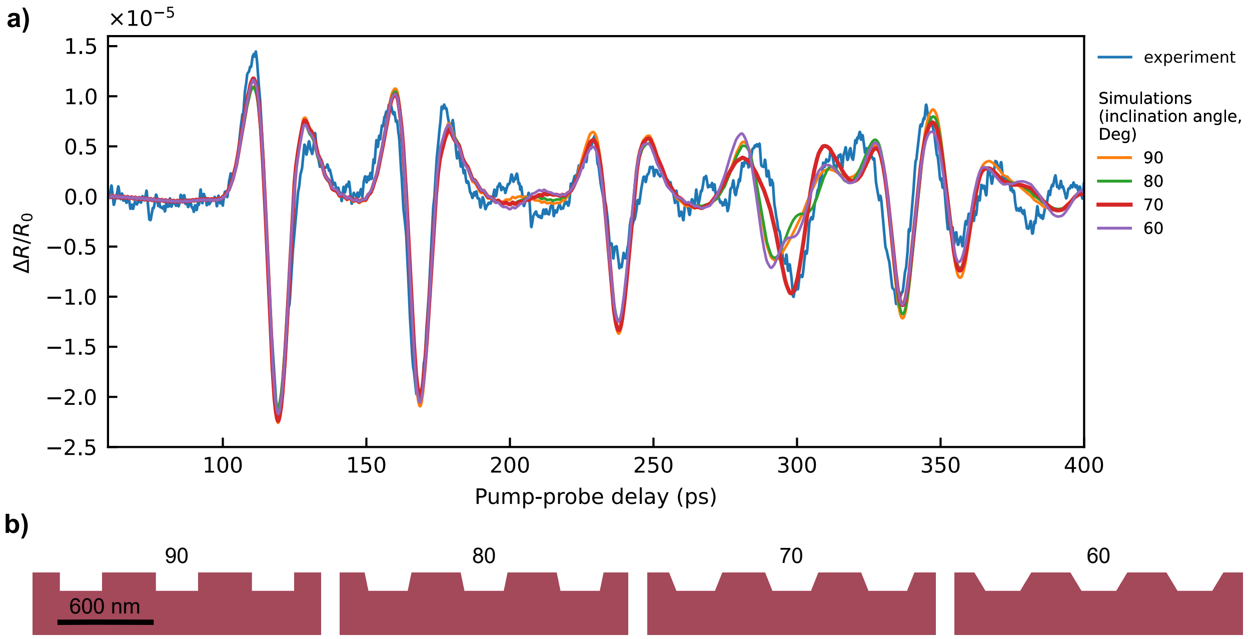

Figure S5: a) Dependence of the time-dependent reflectivity curves on the inclination of grating line walls. The peak associated with acoustic diffraction is most sensitive to small lineshape variations. b) Profiles of grating lines used in simulations.

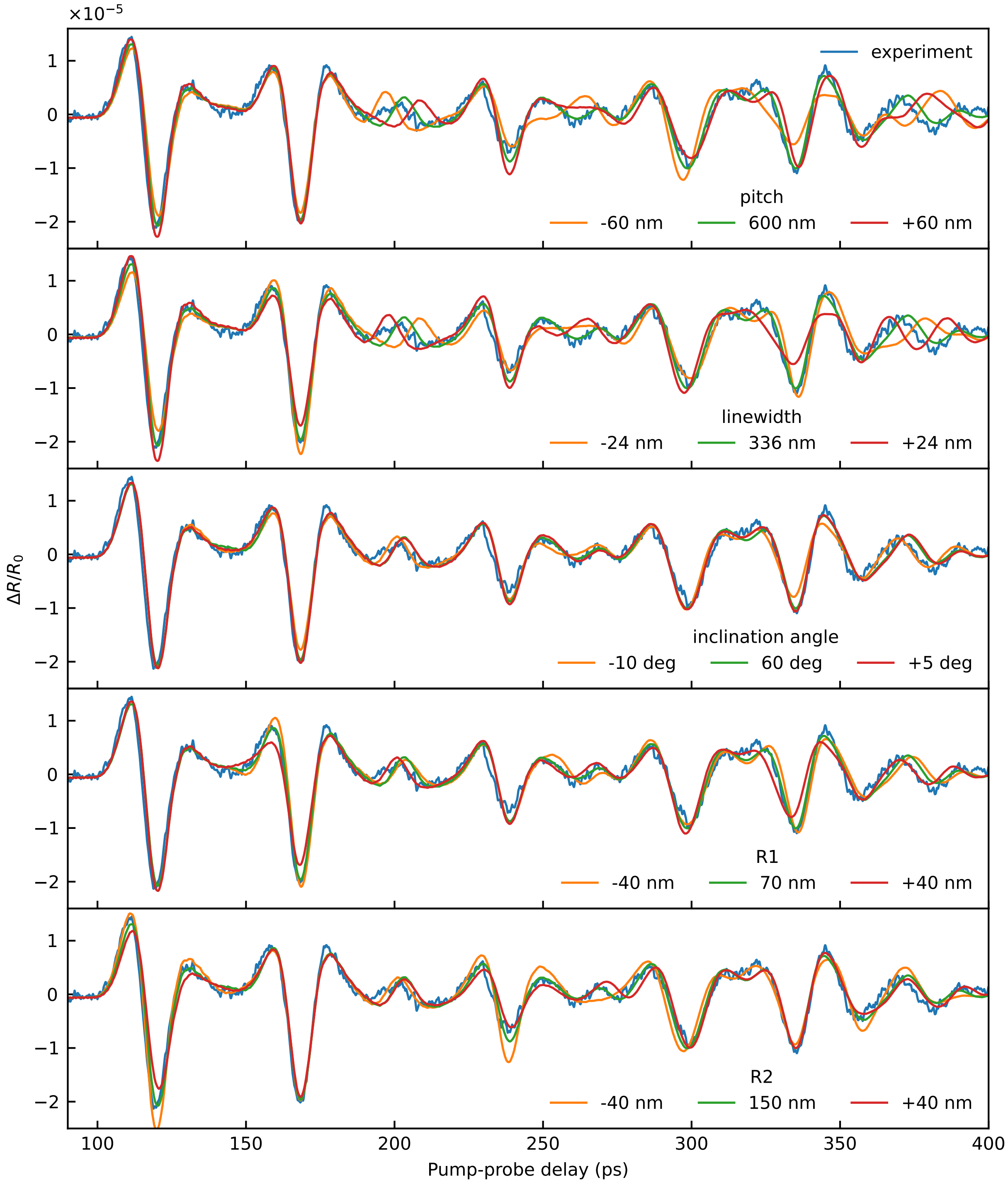

Figure S6: Sensitivity of simulated reflectivity curves of a nominal $600\,\mathrm{nm}$ pitch grating with $300\,\mathrm{nm}$ linewidth to grating parameter variations. Each subplot represents variations in one grating parameter depicted in the legend. Blue curves represent experimental measurements. Green curves show the simulation with optimal grating parameters. Orange and red curves show simulations with deviations in grating parameters from optimal values. Inclination angle is the angle of the sidewalls (where 90 degrees corresponds to a rectangular lineshape). R1 is the radius of curvature of the top corners of the lines, while R2 is the radius of curvature of the bottom corners. See Fig. 4 in the main text for a graphical representation.

the lines. Figure S5 shows the reflectivity curves for different inclination angles. Note that for different inclination angles, the duty cycle of the grating was adjusted (Fig. S5b) to maintain the correct ratio of the amplitudes of the first two echoes. One can see that the inclination of the line walls changes the reflectivity curves. For an inclination of 70 degrees, the shape of the diffraction peak is very close to the one measured in the experiment. However, the mismatch in the amplitude of the echo 3 remains.

The fits in Figs. S4 and S5 show the clear limitations when comparing experimental data with rectangular or trapezoidal lineshapes. As shown in the main text, including the rounded shapes at the top and bottom corners of the grating lines leads to a much more accurate fit to the experimental data. To assess the sensitivity to these structural parameters, Fig. S6 shows the effect of a variation of each parameter separately. In each panel, the green trace represents the best fit to the data, while the red and orange curves show the effect of increasing or decreasing the value of one parameter by an amount that leads to a clear deviation from an optimal fit. From these traces, it can be seen that the sensitivity to each parameter is in the range of 20-60 nm for our Zr samples. The sensitivity to a change in sidewall angles seems to be on the order of 10 degrees, although this parameter is not fully independent of the curvatures and line height: for the present fit, a sidewall angle above 65 degrees would not be realistic, as it would not be possible to parametrize a lineshape with the optimized curvatures, line height and line width using such steep angles. While the multi-parameter nature of the fits makes it challenging to define error bars on each individual parameter, Fig. S6 shows that each parameter has a distinct effect on the overall time-dependent reflectivity signal, giving further confidence in the uniqueness of the fits.

As an alternative, intuitive way to parametrize such rounded gratings, we also considered a sigmoidal lineshape of the form:

$$h = \frac{1}{1 + \exp\left(\frac{x - x_0}{m}\right)} \tag{10}$$

in which x_0 is the center position of the edge, and the parameter m controls the amount of smoothing of the edge (with $m \to 0$ describing a step-like edge, and larger m values correspond to increased smoothing). Simulations with various amounts of curvature are shown in Fig. S7. One can see that the inclination of line walls and the

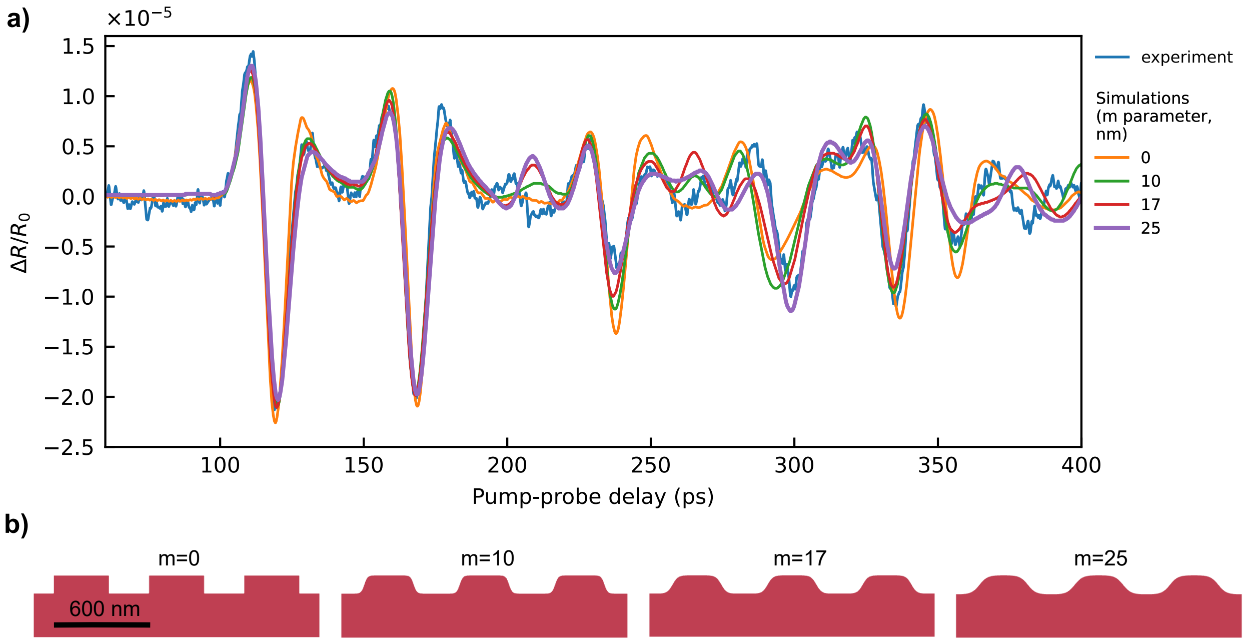

Figure S7: a) Dependence of the time-dependent reflectivity curves on the m parameter of the grating lineshape. Rounded lineshapes give better agreement with experiments than just inclined line walls. b) Profiles of grating lines used in simulations.

rounding of line edges helps both with the time position of the diffraction peak and the amplitude of the peak 3. While these simulations are illustrative, the chosen sigmoidal lineshape links the inclination and roundness of edges, limiting the freedom of fitting. Even better agreement between theory and experiments is achieved (see the main text and Fig. S6) by independently adjusting the inclination and roundness of the edges, although this sigmoidal shape already provides a simple and intuitive lineshape model.

References

[1] C. Thomsen, H. T. Grahn, H. J. Maris, and J. Tauc, "Surface generation and detection of phonons by picosecond light pulses," *Phys. Rev. B*, vol. 34, pp. 4129–4138, Sept. 1986.

[2] P. Ruello and V. E. Gusev, "Physical mechanisms of coherent acoustic phonons generation by ultrafast laser action," *Ultrasonics*, vol. 56, pp. 21–35, Feb. 2015.

[3] M. Kaganov, "Relaxation between electrons and the crystalline lattice," *Sov. Phys. JETP*, vol. 4, no. 2, pp. 173–178, 1957.

[4] S. I. Anisimov, B. L. Kapeliovich, and T. L. Perel'Man, "Electron emission from metal surfaces exposed to ultrashort laser pulses," *Sov. Phys. JETP*, vol. 39, pp. 375–377, Aug. 1974.

[5] T. Q. Qiu and C. L. Tien, "Heat Transfer Mechanisms During Short-Pulse Laser Heating of Metals," *J. Heat Transfer*, vol. 115, pp. 835–841, Nov. 1993.

[6] N. W. Ashcroft and N. D. Mermin, *Solid State Physics*. Holt, Rinehart and Winston, 1976.

[7] Z. Lin, L. V. Zhigilei, and V. Celli, "Electron-phonon coupling and electron heat capacity of metals under conditions of strong electron-phonon nonequilibrium," *Phys. Rev. B*, vol. 77, p. 075133, Feb. 2008.

[8] S. J. Byrnes, "Multilayer optical calculations," Dec. 2020.

[9] L. D. Landau, E. M. Lifshitz, A. M. Kosevich, and L. P. Pitaevskii, *Theory of Elasticity: Volume 7*. Elsevier, Jan. 1986.

[10] D. Li and D. G. Cahill, "Attenuation of 7 GHz surface acoustic waves on silicon," *Phys. Rev. B*, vol. 94, p. 104306, Sept. 2016.

[11] A. Devos, M. Foret, S. Ayrinhac, P. Emery, and B. Rufflé, "Hypersound damping in vitreous silica measured by picosecond acoustics," *Phys. Rev. B*, vol. 77, p. 100201, Mar. 2008.

[12] B. C. Daly, K. Kang, Y. Wang, and D. G. Cahill, "Picosecond ultrasonic measurements of attenuation of longitudinal acoustic phonons in silicon," *Phys. Rev. B*, vol. 80, p. 174112, Nov. 2009.

[13] H. Zhang, A. Antoncecchi, S. Edward, I. Setija, P. Planken, and S. Witte, "Unraveling Phononic, Optoacoustic, and Mechanical Properties of Metals with Light-Driven Hypersound," *Phys. Rev. Appl.*, vol. 13, p. 014010, Jan. 2020.

[14] M. C. Velsink, M. Illienko, P. Sudera, and S. Witte, "Optimizing pump–probe reflectivity measurements of ultrafast photoacoustics with modulated asynchronous optical sampling," *Rev. Sci. Instrum.*, vol. 94, p. 103002, Oct. 2023.

[15] P. A. Elzinga, F. E. Lytle, Y. Jian, G. B. King, and N. M. Laurendeau, "Pump/Probe Spectroscopy by Asynchronous Optical Sampling," *Appl. Spectrosc.*, vol. 41, pp. 2–4, Jan. 1987.

[16] D. E. Gray, *American Institute of Physics Handbook: 3d Ed.* McGraw-Hill, 1972.

[17] D. R. Lide, *CRC Handbook of Chemistry and Physics*, vol. 85. CRC press, 2004.

[18] M. R. Querry, *Optical Constants of Minerals and Other Materials from the Millimeter to the Ultraviolet*. CRDEC-CR-88009, Aberdeen Proving Ground, Maryland, 1987.
